# Antibacterial Activities and *In Vitro* Anti-Inflammatory (Membrane Stability) Properties of Methanolic Extracts of *Gardenia coronaria* Leaves

**DOI:** 10.1155/2014/410935

**Published:** 2014-02-19

**Authors:** Amin Chowdhury, Shofiul Azam, Mohammed Abdullah Jainul, Kazi Omar Faruq, Atiqul Islam

**Affiliations:** Department of Pharmacy, International Islamic University Chittagong, College Road, Chawkbazar, Chittagong 4203, Bangladesh

## Abstract

This work is carried out with *Gardenia coronaria* leaves that belong to the family Rubiaceae, which is a small-to-medium-sized but tall, deciduous tree, 7.6–9 m high on an average. Leaves are used for the treatment of rheumatic pain and bronchitis. The leaf of the plant consists of coronalolide, coronalolic acid, coronalolide methyl ester, ethyl coronalolate acetate triterpenes (*secocycloartanes*), and so forth. Methanol extract from the leaves of *Gardenia coronaria* was completely screened for membrane stability and antibacterial activity. The lower concentrations of Methanolic leaf extract of *Gardenia coronaria* gave good antimicrobial and anti-inflammatory activity, but higher concentrations gave relatively more projecting antibacterial activity *in vitro* as compared with Kanamycin. The crude drug's anti-inflammatory effects were compared with those of Aspirin as positive control. The Methanolic extracts of *Gardenia coronaria* leaves possessed a broad spectrum antibacterial activity against a variety of both Gram-negative and Gram-positive organisms like *Streptococcus agalactiae*, *Escherichia coli*, *Pseudomonas aeruginosa*, *Bacillus cereus*, *Shigella sonnei*, *Shigella boydii*, and *Proteus mirabilis*, with a zone of inhibition from 10 to 16 mm. The extract also showed good membrane stability to be considered as having significant anti-inflammatory action.

## 1. Introduction

From the very beginning of the civilization, there is an extreme relationship between human beings and plants. In ancient period the system of treatment was not enriched like today. The ancient people used to utilize several parts of plants in different treatment purposes. Plants were not only used as medicine, but also in a number of their daily jobs (e.g., fishing, hunting, etc.) purposes. Eventually, plants are the ultimate caretaker of environment in a sense. A single part of plant may consist of numerous medicinal values, but it has been proved that direct intake of crude plant is not good, as it contains both essential and nonessential components. The nonessential ones may not be required by the body in healing purposes or in other circumstances; the nonessential components may in fact be toxic to the body under some cases. Even the intake of the essential components via the crude extract may lead to an improper dose [1].

An antibacterial agent that either kills microorganism or suppresses its growth is often termed as antibiotic. The term antibiotic covers a broad range of agents like antimicrobials, including antifungal and other compounds [2]. The term antibiotic was first used by Waksman in 1942; he used the term to describe any substance that antagonizes the growth or kills other microorganisms at high dilution [3]. By the development of modern science, most of today's antibiotics are chemical modification of the 1st generation antibiotics that used to be natural compounds, for example, *Penicillin*, *Cephalosporin*, *Sulfonamide*, *Quinolone*, and so forth [4].

Plant chemicals that are usually supposed to be responsible for antibacterial effects used to have phenolic ring, alkaloid, tannins, and so forth, most commonly. For example, common herbs *thyme* and *tarragon* possess effective antibacterial, antifungal, and antiviral activities, containing caffeic acid in phytochemical list [5–8]. The mechanisms are yet not clear but thought to be responsible for phenolic toxicity to microorganisms via enzyme inhibition by the oxidized compounds, possibly through reaction with sulfhydryl groups or through more nonspecific interactions with the proteins [9].

The control of bacterial infection has been remarkably effective since the discovery of antibacterial drugs. However, some of the pathogens rapidly became resistant to many of the first discovered effective drugs. The development of drug resistance, as well as appearance of undesirable side effects of certain antibiotics [10], has led to the search for new antibacterial agents in particular from medicinal plants. Higher plants have been shown to be a potential source for new antimicrobial agents [11].

Inflammation is one common and major cause of sufferings now and every time past. Those drugs that are available are known as NSAID, that is, nonsteroidal anti-inflammatory drugs, act by inhibiting the function of prostaglandin. Prostaglandin is an autocoid that is released extracellularly and initiate pain. Anti-inflammatory agents block this autocoid synthesis by either inhibiting COX enzyme or protecting lysosomal membrane from breakdown. The leaves of the plant *Gardenia coronaria* are used for the treatment of rheumatic pain and bronchitis by the local people [12].

## 2. Materials and Methods

### 2.1. Plant Material

The plant was collected from Bandarban, the hilly region of the Chittagong hill tracts of Bangladesh, in October 2012. Then the plant was identified by Dr. Shaikh Bokhtear Uddin, Associate Professor, Department of Botany, University of Chittagong. The plant sample, which included a meter in length branch and leaves with an unripe fruit in it, was preserved in a herbarium and was further taken to the Bangladesh Forest Research Institute (BFRI), and again identified to be *Gardenia coronaria* by the plant taxonomist Mr. Sohel.

### 2.2. Extraction

Extraction of plant leaves was done by using organic solvent [13]. The fresh leaves of *Gardenia coronaria* were cut, washed, and air-dried at room temperature (24° ± 2°C) for about 10 days. Dried leaves were milled into coarse powder. Dried powder weighing about (500 grams) was then macerated in Methanol. Then Methanolic mix of the leaf powder was shaken by a rotary shaking apparatus for 7 days. The extract was collected using Buckner funnel, where the Methanolic mix of the powder was poured under vacuum suction. The filtrate contained the crude drug extract of Methanol. The Methanol was evaporated at a temperature below 45°C and a concentrated crude drug of Methanolic extract of *Gardenia coronaria* leaves was obtained, which was weighed to be 29 grams and was poured into an alpine tube and stored at 4°C in the refrigerator.

### 2.3. Antibacterial Screening

#### 2.3.1. Plant Extracts Dilution and Preparation of Impregnated Disc

Stock solution was prepared by dissolving 10 milligrams of the Methanolic crude drug extract in Methanol. Five filter papers were taken and punched to uniform discs sized 6 mm in diameter each. Sterilized filter paper discs were taken with a sterile forceps in the plates. Sample solutions of desired concentrations (100 *μ*g/disc, 200 *μ*g/disc, 400 *μ*g/disc, and 500 *μ*g/disc) were applied in the disc with the help of the micropipette in an aseptic condition. These discs were left for a few minutes in aseptic condition for complete evaporation of the solvent. Standard discs were used to compare the antibacterial activity of the test material. In the present study, Kanamycin disc was prepared with injecting the Kanamycin sulfate-injectable dosage form (brand name Kantrex) on a disc. K-30 discs containing 30 *μ*g/disc of antibiotic Kanamycin were used as a standard disc for comparison purpose. The *in vitro* disc diffusion assay method [14] of antibacterial screening was used to determine the susceptibility of the pathogenic microorganisms to the test compound applied. The plate diffusion test utilizes different concentrations of a test compound absorbed on sterile filter paper disks on the same plate containing a specific organism.

#### 2.3.2. Test Organism Used for the Study and Sterilization Procedures

Seven pathogenic bacteria were selected for the test, of which two were Gram-positive and the remaining five were Gram-negative organisms. These organisms of pure culture were collected from the Microbiology Lab, International Islamic University Chittagong; the bacterial strains used for this investigation are listed in Table 1.

The antibacterial screening was carried out in a laminar air flow unit and all types of precautions were highly maintained to avoid any type of contamination during the test. Ultraviolet light was switched on for half an hour before working in the laminar hood to avoid any accidental contamination. Petri dishes and other glassware were sterilized in the autoclave at 121°C temperature and a pressure of 15 lbs/sq inch for 15 minutes. Micropipette tips, culture media, cotton, forceps, blank disks, and so forth, were also sterilized [15].

#### 2.3.3. Preparation of Nutrient Agar Medium, Fresh Culture of the Pathogenic Organisms, Test Plates, and Placement of Impregnated Discs on Test Plates

The instant nutrient agar medium (Difco) was weighed and then reconstituted with distilled water in a conical flask according to specification (2.3% w/v). It was then heated in a water bath to dissolve the agar until a transparent solution was obtained [16].

The nutrient agar medium was prepared and dispersed in a number of test tubes to prepare slants (5 mL in each test tube). This was done to prepare axenic cultures from the supplied cultures [17, 18]. The test tubes were plugged with cotton and sterilized in the autoclave at 121°C temperature and a pressure of 15 lbs/sq inch for 15 minutes. After sterilization, the test tubes were kept in an inclined position for solidification. These were then incubated at 37.5°C to be sure that they were sterile. The test organisms were transferred to the agar slants from the supplied cultures with the help of an inoculating loop in aseptic condition. The loop was burned after each transfer of microorganism to avoid contamination very carefully. The culture was kept at 4°C or less for bacterial growth for 12 hours and then incubated at 37°C for 24 hours to assure the growth of test organisms. These fresh axenic cultures were then used for the sensitivity test.

The test plates were prepared for the disc diffusion test of the test samples [19]. At first, the nutrient agar medium prepared previously was poured in 15 mL quantity in each of the clean test tubes and plugged with the cotton. The test tubes and a number of Petri dishes for this purpose were sterilized in an autoclave at 121°C temperature and a pressure of 15 lbs./sq. inch for 15 minutes and were transferred into a laminar air flow unit and then allowed to cool for 45 to 50°C. The test organisms were transferred from the fresh subculture to the test tube containing 15 mL autoclaved medium with the help of an inoculating loop in an aseptic condition. Then the test tube was shaken by rotation to get a uniform suspension of the organism. Bacterial suspensions were then immediately transferred to the sterile Petri dishes in an aseptic area. The Petri dishes were rotated several times, first clockwise and then anticlockwise to assure homogenous distribution of the test organisms. The medium was poured into Petri dishes in such a way, in order to give a uniform depth of approximately 4 mm.

Finally, the medium was cooled to room temperature in laminar air flow unit and it was kept in refrigerator at 4°C and the sample impregnated discs and standard disc were seeded, in the subsolidified medium. The medium was congealed to room temperature in laminar air flow unit and then refrigerated at 4°C for 24 hours in order to provide sufficient time to diffuse the antibiotics into the medium. The plates were incubated at 37°C for 24 hours in an incubator and, after incubation, the antibacterial activities of the test samples were determined by measuring the diameter of inhibitory zones. Hence, the zones of inhibition of different samples were compared [20].

## 3. Anti-Inflammatory (Membrane Stability) Activity Assay

### 3.1. Method and Principle

The lysosomal enzyme released during inflammation produces a variety of disorders. The extracellular activity of these enzymes is said to be related to acute or chronic inflammation. The nonsteroidal drugs act either by inhibiting these lysosomal enzymes or by stabilizing the lysosomal membrane. Since HRBC (human red blood cell) membrane is similar to lysosomal membrane, the study was undertaken to check the stability of HRBC membrane by the extracts to predict the anti-inflammatory activity *in vitro*. The various extracts at the concentration of 100, 200, and 300 *μ*g/mL, respectively, were incubated separately with HRBC solution [21].

### 3.2. Procedure

#### 3.2.1. HRBC Membrane Stabilization Method

The anti-inflammatory activity of various extracts of leaves of *Gardenia coronaria* was assessed by *in vitro* HRBC membrane stabilization method. Blood was collected from healthy volunteers. The collected blood was mixed with equal volume of Alsever solution (dextrose 2%, sodium citrate 0.8%, citric acid 0.05%, sodium chloride 0.42%, and distilled water 100 mL) and centrifuged with isosaline. To 1 mL of HRBC suspension, equal volume of test drug in three different concentrations, 100, 200, and 300 *μ*g/mL, was added. All the assay mixtures were incubated at 37°C for 30 minutes and centrifuged. The haemoglobin content in the supernatant solution was estimated by using spectrophotometer at 560 nm [22]. The percentage of haemolysis was calculated then by the formula as given below:

(1) $$\begin{array}{c} \text{Percent}\;\text{of}\;\text{hemolysis}=\frac{\text{OD}\;\text{of}\;\text{test}}{\text{OD}\;\text{of}\;\text{control}}\times 100. \end{array}$$

The percentage of protection can be hence calculated from the equation as given below:

(2) $$\begin{array}{c} \text{Percent}\;\text{of}\;\text{protection}=100-\frac{\text{OD}\;\text{of}\;\text{test}}{\text{OD}\;\text{of}\;\text{control}}\times 100. \end{array}$$

Here “OD of test” is optical density or the test sample's absorbance and “OD of control” is optical density or absorbance of the negative control.

Here, the negative control used was Alsever's solution with blood in it and it contained no Aspirin or Methanolic extract of the plant material in it. The absorbance of the negative control was found to be 0.242 (Table 3).

## 4. Results and Discussion

The antimicrobial activity of the Methanolic extract of leaves of *Gardenia coronaria* was measured by disc diffusion method. Different concentrations of 100 *μ*g/disc, 200 *μ*g/disc, 400 *μ*g/disc, and 500 *μ*g/disc were measured and compared with the zone of inhibitions with that produced by the standard antibiotic, Kanamycin at 30 *μ*g/disc.

**Figure 1:**
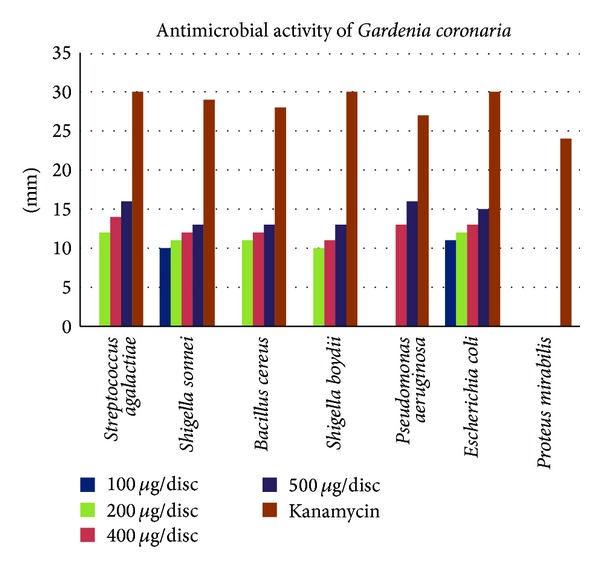
Graphical representation of difference in zone of inhibition of different bacteria in different extract concentrations of *Gardenia coronaria* leaves along with the standard Kanamycin.

The zones of inhibition were seen against selective bacteria at a particular concentration (Table 2). The studied Methanolic extract of leaves of plant *Gardenia coronaria* showed higher activity against *E*. *coli*, *Shigella sonnei*, and *Streptococcus agalactiae*. At higher concentrations of 400 *μ*g/disc and 500 *μ*g/disc, the extract also showed good inhibitions against *Pseudomonas aeruginosa* of 13 mm and 16 mm, respectively. However, the extract showed no activity against the Gram-negative bacteria *Proteus mirabilis* (Figure 1).

**Figure 2:**
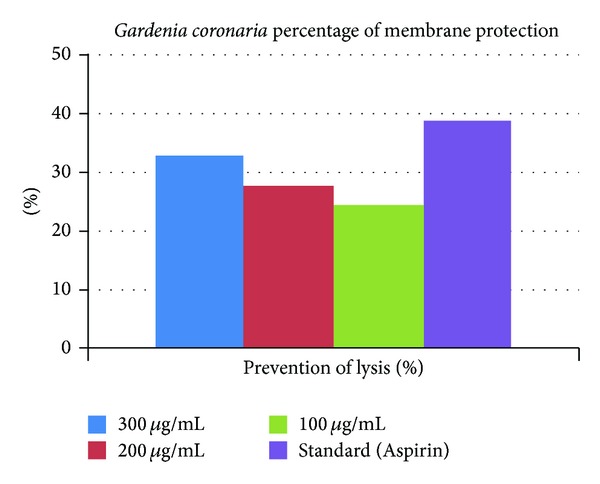
The bar chart above represents the percentage of membrane protection of the Methanolic leaves extract with a reference to Aspirin 100 *μ*g/mL as standard.

The extract at concentration range from 100 *μ*g/mL to 300 *μ*g/mL protects the human erythrocyte membranes against lysis induced by hypotonic solution. At concentration of 100 *μ*g/mL, the extract inhibited 24.38% of RBC haemolysis as compared with 38.84% produced by Aspirin at 100 *μ*g/mL (Figure 2). Since human red blood cell membranes are similar to lysosomal membrane components, the prevention of hypotonicity induced HRBC membrane lysis was taken as a measure of anti-inflammatory activity of drugs. The results obtained demonstrated that Methanolic extract of leaves of *Gardenia coronaria* can significantly and dose dependently inhibit HRBC haemolysis.

This *in vitro* method was more time saving, flexible, and convenient in other ways. The investigation suggested good ability of the Methanolic leaves extract to resist the cell lysis in small concentrations as compared to the standard drug Aspirin at 100 *μ*g/mL, though not greater than Aspirin. Even the highest concentration of the extract at 300 *μ*g/mL was able to prevent lysis of 33% which was yet 5.84% less than that of Aspirin, when used in only 100 *μ*g/mL (Figure 2). From the study of this experiment, it may be concluded that the Methanolic extract of the leaves of *Gardenia coronaria* plant has good membrane stability, hence good anti-inflammatory activities. Since the Methanolic extract of leaves of *Gardenia coronaria* shows significant antibacterial and anti-inflammatory properties, further laboratory study and chemical isolation of this plant leaves might confirm an effective drug molecule in pharmacologic aspects effectively, in both types of pharmaceutical arena.

## Figures and Tables

**Table 1:** The list of pathogenic bacteria used in the test.

Serial number	Name of the test organism
Gram-positive	
1	*Streptococcus agalactiae *
2	*Bacillus cereus *

Gram-negative	
3	*Shigella sonnei *
4	*Shigella boydii *
5	*Pseudomonas aeruginosa *
6	*Escherichia coli *
7	*Proteus mirabilis *

**Table 2:** The zones of inhibition against selective bacteria by Methanolic extract of *Gardenia coronaria* leaves.

Name of the bacteria	Zone of inhibition				
	Kanamycin disc (30 *μ*g/disc) (mm)	*Gardenia coronaria* (Methanol extract)			
		100 *μ*g/disc (mm)	200 *μ*g/disc (mm)	400 *μ*g/disc (mm)	500 *μ*g/disc (mm)
*Streptococcus agalactiae *	30 mm	—	12	14	16
*Shigella sonnei *	29 mm	10	11	12	13
*Bacillus cereus *	28 mm	—	11	12	13
*Shigella boydii *	30 mm	—	10	11	13
*Pseudomonas aeruginosa *	27 mm	—	—	13	16
*Escherichia coli *	30 mm	11	12	13	15
*Proteus mirabilis *	24 mm	—	—	—	—

Here, (—) mm indicates no zone of inhibition.

**Table 3:** Data representing absorbance and percentage prevention of lysis.

Concentration (*μ*g/mL)	Absorbance	Prevention of lysis (%)
300	0.162	33
200	0.175	27.68
100	0.183	24.38
Aspirin	0.148	38.84
Negative control	0.242	0
